# Brd4 Is Displaced from HPV Replication Factories as They Expand and Amplify Viral DNA

**DOI:** 10.1371/journal.ppat.1003777

**Published:** 2013-11-21

**Authors:** Nozomi Sakakibara, Dan Chen, Moon Kyoo Jang, Dong Wook Kang, Hans F. Luecke, Shwu-Yuan Wu, Cheng-Ming Chiang, Alison A. McBride

**Affiliations:** 1 Laboratory of Viral Diseases, National Institute of Allergy and Infectious Diseases, National Institutes of Health, Bethesda, Maryland, United States of America; 2 Laboratory of Bioorganic Chemistry, National Institute of Diabetes and Digestive and Kidney Diseases, National Institutes of Health, Bethesda, Maryland, United States of America; 3 Simmons Comprehensive Cancer Center, University of Texas Southwestern Medical Center, Dallas, Texas, United States of America; 4 Department of Biochemistry, University of Texas Southwestern Medical Center, Dallas, Texas, United States of America; 5 Department of Pharmacology, University of Texas Southwestern Medical Center, Dallas, Texas, United States of America; University of Michigan, United States of America

## Abstract

Replication foci are generated by many viruses to concentrate and localize viral DNA synthesis to specific regions of the cell. Expression of the HPV16 E1 and E2 replication proteins in keratinocytes results in nuclear foci that recruit proteins associated with the host DNA damage response. We show that the Brd4 protein localizes to these foci and is essential for their formation. However, when E1 and E2 begin amplifying viral DNA, Brd4 is displaced from the foci and cellular factors associated with DNA synthesis and homologous recombination are recruited. Differentiated HPV-infected keratinocytes form similar nuclear foci that contain amplifying viral DNA. We compare the different foci and show that, while they have many characteristics in common, there is a switch between early Brd4-dependent foci and mature Brd4-independent replication foci. However, HPV genomes encoding mutated E2 proteins that are unable to bind Brd4 can replicate and amplify the viral genome. We propose that, while E1, E2 and Brd4 might bind host chromatin at early stages of infection, there is a temporal and functional switch at later stages and increased E1 and E2 levels promote viral DNA amplification, displacement of Brd4 and growth of a replication factory. The concomitant DNA damage response recruits proteins required for DNA synthesis and repair, which could then be utilized for viral DNA replication. Hence, while Brd4 can enhance replication by concentrating viral processes in specific regions of the host nucleus, this interaction is not absolutely essential for HPV replication.

## Author Summary

Papillomaviruses have a remarkable infection cycle that depends on the development of a stratified epithelium. The virus infects the lower, dividing layers of the epithelium and viral genomes replicate at low copy number, and are maintained in these cells, for long periods of time. As infected cells differentiate and move to the surface of the epithelium, they switch on high level viral DNA replication, synthesize capsid proteins and form new viral particles. Viral replication takes place in nuclear foci and is dependent on the E1 and E2 replication proteins. Brd4 is a cellular chromatin binding protein that interacts with E2 and is important for transcriptional regulation of papillomaviruses. In this study we examine the role of Brd4 at different stages in the formation of viral replication foci. In the absence of viral DNA replication, Brd4 links the viral proteins to host chromatin. However, when viral genomes begin to amplify to high levels, Brd4 is displaced from nuclear foci and is not required for replication.

## Introduction

Papillomaviruses have a complex life cycle in which viral DNA replication is tightly linked to host cell differentiation. Papillomaviruses initially infect the basal cells of the host epithelium and establish a long-term persistent infection in which the viral genome is maintained as an extrachromosomal replicon in the dividing cells. As the infected cells differentiate, replication switches to a vegetative mode to produce large numbers of progeny genomes [1]. There are thought to be three different modes of replication in this life cycle. The first is the initial burst of amplificational replication that occurs when a virion genome is delivered to the dividing host cell and becomes established as a low copy number extrachromosomal plasmid. The second phase is when these established genomes are replicated and partitioned along with host cellular DNA in proliferating cells. For many papillomaviruses, the E2 replication protein maintains and partitions the genomes by tethering them to mitotic chromosomes, often in complex with the chromatin binding protein, Brd4 (reviewed in [2], [3]. Finally, the third phase occurs when persistently infected cells differentiate and the cells begin to express high levels of the E1 and E2 replication proteins and large numbers of progeny viral genomes are synthesized.

We, and others, have previously shown that expression of HPV E1 and E2 proteins results in the formation of nuclear foci that recruit DNA damage response proteins and show evidence of DNA synthesis [4]–[6]. The replication process and/or replication proteins of many viruses activate a DNA damage response, but many viruses exploit this response to enhance their own replication [7]. We have proposed that the HPV E1/E2 foci represent viral replication foci and have hypothesized that HPV replication induces a cellular DNA damage response, but the virus takes advantage of this to recruit components required for DNA synthesis to specific nuclear foci in differentiated cells [4], [8]. Studies using keratinocytes naturally infected with HPV31 demonstrate that the ATM DNA damage response enhances differentiation dependent amplification of the viral genome [9] and this occurs in replication centers that recruit DNA damage markers as well as markers of DNA repair [10].

In our previous study we defined the functions of the E1 and E2 proteins that are required for the formation of nuclear foci and induction of the DNA damage response [4]. The origin specific binding and ATPase functions of the E1 protein were necessary for induction of the ATM/ATR pathways as well as for the formation of foci. Therefore, the foci are not simply due to interaction and colocalization of the E1 and E2 proteins in the nucleus, since they require specific activities of the replication initiator helicase. The E2 protein was not required for the induction of the DNA damage response, but it was necessary for the formation of the nuclear foci. As expected, a mutation that disrupts the interaction of the E1 and E2 proteins abrogated the nuclear E1–E2 foci, while the DNA binding function of E2 was not required. However, a mutation (R37A/I73A) that specifically inactivated the transcriptional regulatory function of E2 also abrogated the formation of nuclear E1–E2 foci. These residues are crucial for interaction of the E2 protein with the host Brd4 protein [11]–[13] and this led us to examine the role of Brd4 in the formation of papillomavirus replication foci, as well as in the processes of papillomavirus DNA replication.

In this study we examine the relationship and requirement of Brd4 within viral replication foci. We find that Brd4 is required for the formation of small early foci formed by the E1 and E2 proteins. However, when the genome begins to amplify in the replication foci there is a temporal switch of E2 function and Brd4 is not essential for the replication function of E2 and is not required for the amplification of these foci.

All papillomavirus E2 proteins bind to the cellular Brd4 protein to regulate viral transcription [14]–[17], but only the E2 proteins from a subset of viruses bind Brd4 with high affinity and stabilize the interaction of Brd4 with chromatin [14], [18]. These E2 proteins also readily bind to mitotic chromosomes and this is thought to be important for viral genome tethering and partitioning [19], [20]. E2 proteins from the alpha genus of papillomaviruses (a group of viruses that infect primarily the mucosal epithelium and contain the oncogenic HPVs) do not bind tightly to host chromatin and are not easily detected on mitotic chromosomes. In this study we also show that in the presence of the homologous E1 protein, the oncogenic, alpha-HPV16 E2 protein binds tightly to regions of host chromatin enriched for Brd4.

## Results

### The Brd4 protein localizes to E1–E2 nuclear foci

**Figure 1:**
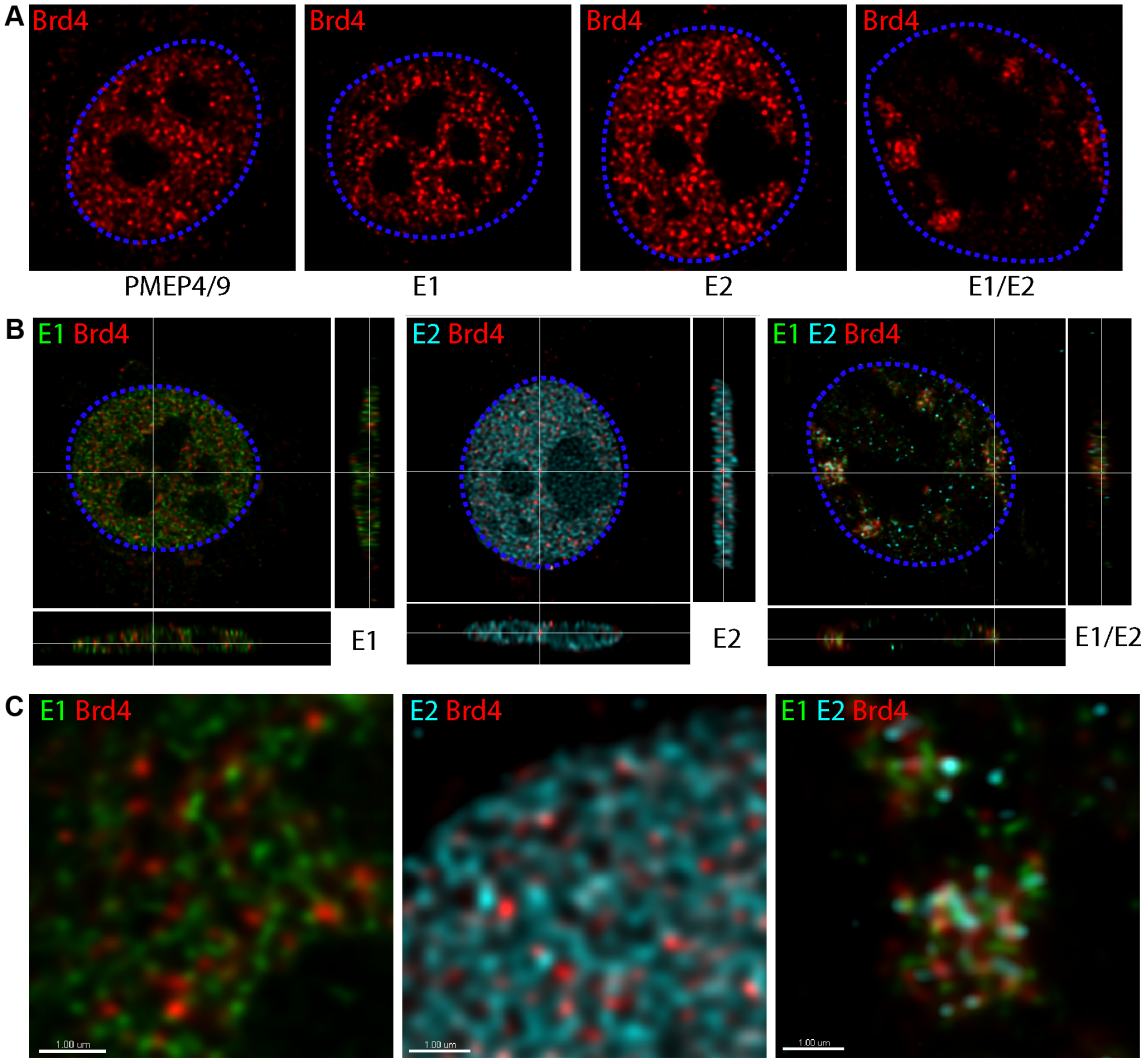
Brd4 localizes to nuclear foci formed by HPV16 E1 and E2 proteins. Human keratinocytes were transfected with pMEP4/9 (empty), pMEP9-HPV16 E1 and pMEP4-HPV16 E2 expression vectors, as indicated. E1, E2 and endogenous Brd4 proteins were detected by indirect immunofluorescence. Staining for E1 proteins is shown in green, E2 proteins in cyan, and Brd4 in red. High resolution 3D images reconstructed by deconvolution of stacks of confocal images are shown. At least five cells were digitally reconstructed from each condition and a representative image is shown. The dashed blue lines represent the perimeter of the nuclei (identified by DAPI staining, not shown). **A.** Brd4 staining in a single optical slice through the center of the nucleus. **B.** An optical section through nuclei expressing E1, E2 or E1/E2. The crosshairs show the cut point through the x-, y-, and z-axes of the data set. These are the same cells shown in Figure 1A. **C.** An enlargement of a portion of the nuclei from the images shown in Figure 1B. The scale bar represents 1 µm.

Our previous study showed that mutation of residues R37 and I73 in the HPV16 E2 protein abrogated its ability to form E1–E2 nuclear foci [4]. Since these residues are important for the interaction of E2 and Brd4, we investigated whether the Brd4 protein co-localized with the nuclear E1/E2 foci. Human keratinocytes were cotransfected with expression vectors for the HPV16 E1 and E2 proteins. As shown previously, this led to the appearance of nuclear foci containing both proteins, as detected by immunofluorescence, in about 50% of E1 expressing cells. In the absence of E1 or E2, or when each protein was expressed individually, Brd4 was observed as small speckles distributed throughout the nucleus. However, when E1 and E2 were coexpressed, there was a complete reorganization of Brd4 (Figure 1A) and Brd4 was observed concentrated in the E1/E2 foci (Figures 1B and C). A similar enrichment in the apparent colocalization of E1, E2 and Brd4 was also observed in CV1 and C-33A cells (data not shown).

The representative images shown in Figures 1B and C were generated by 3D reconstruction of stacks of confocal images that were deconvolved to remove out of focus blur and improve colocalization analysis. When expressed alone, each viral protein was distributed in a fine, granular pattern throughout the nucleus that showed only very minimal colocalization with the Brd4 speckles (Figures 1B and C). In contrast, coexpression of E1 and E2 resulted in large nuclear foci that contained concentrated Brd4. However, high resolution 3D analysis showed that each focus consisted of a cluster of tiny E1, E2 and Brd4 speckles and did not represent a complete colocalization of the three proteins.

### Salt extraction of cells expressing HPV16 E1 and E2 show that the E1 protein binds most tightly to host nuclei

Previously, our laboratory had analyzed the interaction of a series of E2 proteins with Brd4 and found that, while most E2 proteins co-localized with Brd4 in interphase and mitotic chromatin, the alpha-papillomaviruses (including HPV16 E2) did not [14]. Furthermore, both Brd4 and the alpha-papillomavirus E2 proteins were easily eluted from the host nucleus by low salt extraction, indicating that they were not tightly associated with host chromatin [14]. We show here that, in the presence of the homologous E1 protein, there is very specific clustering of alpha-E2 and Brd4 in nuclear foci.

**Figure 2:**
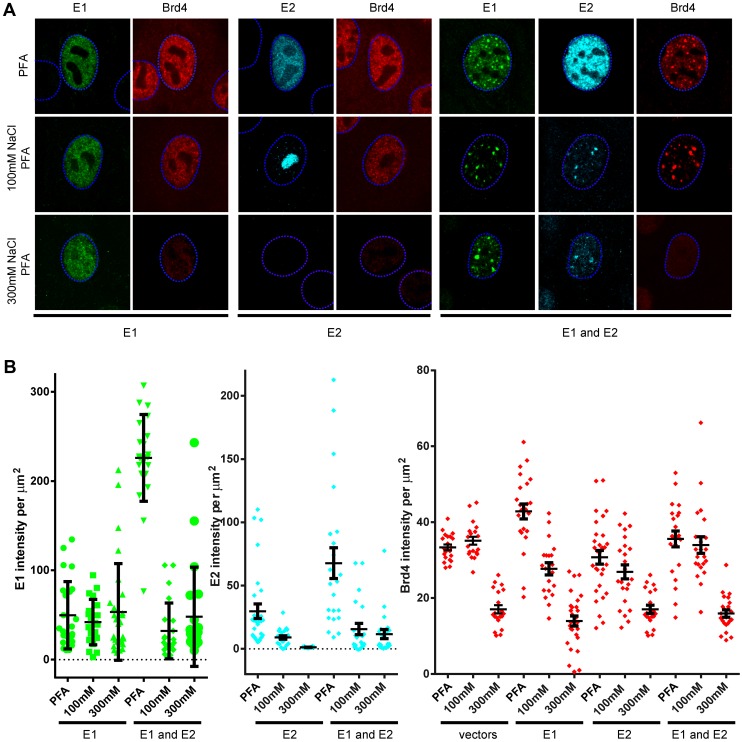
Salt extraction of cells expressing HPV16 E1 and E2 show that the E1 protein binds most tightly to host nuclei. Human keratinocytes were cotransfected with expression vectors for HPV16 E1 (pMEP9-E1), HPV16 E2 (pMEP4-E2), or both proteins. Cells were either fixed directly in paraformaldehyde (PFA), or proteins were extracted from cells in buffers containing either 100 mM or 300 mM NaCl prior to fixation in PFA. **A.** E1, E2 and endogenous Brd4 proteins were detected by indirect immunofluorescence. Staining for the E1 protein is shown in green, the E2 protein in cyan, and Brd4 in red. The dashed blue lines represent the perimeter of the nuclei (identified by DAPI staining, not shown). **B.** Level of E1, E2, and Brd4 were measured in individual cells extracted and fixed as described above using the Contour feature of Imaris software (Bitplane) to extract values from each channel in individual nuclei. Average values obtained from at least five reconstructed nuclei are shown and the error bars represent the standard deviation. Individual transfected cells could not be identified in cells transfected with the empty pMEP4/9 vectors or in cells transfected with HPV16 E2 vectors and extracted in 300 mM NaCl containing buffer. In these samples, random cells were selected for quantitation.

To further investigate the association of HPV16 E1, E2 and Brd4 with host chromatin, transfected keratinocytes were pre-extracted with 100 mM or 300 mM salt before fixation and immunofluorescence. As shown in Figure 2A and B, when expressed alone the E1 protein was resistant to salt extraction but, as observed previously [14], the HPV16 E2 protein was not. In fact, after extraction in 100 mM NaCl, E2 was found in the nucleolus, and after 300 mM extraction E2 was undetectable. However, when the E1 and E2 proteins were co-expressed, the E1–E2 complex was tightly bound to chromatin. This implies that this association is primarily mediated by the E1 protein. Further pre-extraction of keratinocytes with 300 mM salt removes Brd4 (in particular the bright Brd4 foci, as observed in Figure 1A) from the nucleus in the presence or absence of E1 and E2, leaving behind the E1–E2 foci. Therefore, this association is different from the tightly bound E2-Brd4 complex observed with non-alpha papillomaviruses [14].

### Brd4 is displaced from E1–E2 Foci in the presence of an HPV replicon

**Figure 3:**
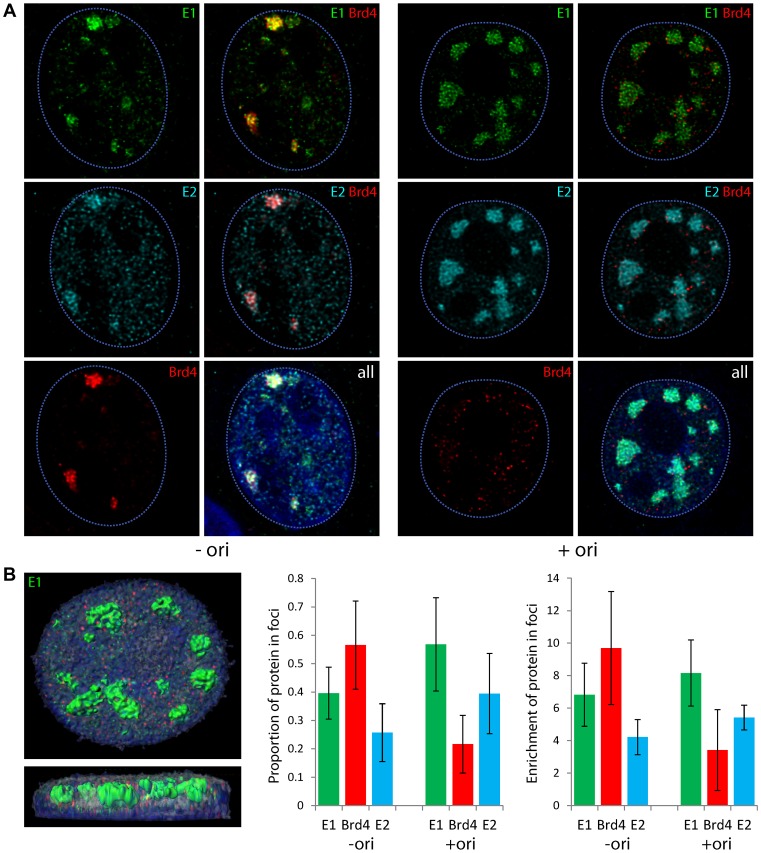
Localization of Brd4 to E1–E2 replication foci changes in the presence of the replication origin. Human keratinocytes were cotransfected with expression vectors for HPV16 E1 and E2 in the absence or presence of a plasmid containing the minimal HPV16 replication origin, p16ori. **A.** In the panels on the left, keratinocytes were transfected without the origin plasmid (instead the control plasmid pKS bluescript was used) and on the right in the presence of the origin. High resolution 3D images were reconstructed by deconvolution of stacks of confocal images and a representative optical slice is shown for a representative cell with and without the origin. Staining for E1 proteins is shown in green, E2 proteins in cyan, and the Brd4 protein in red. The dashed blue lines represent the perimeter of the nuclei (identified by DAPI staining, not shown). **B.** To quantitate the amount of E1, E2 and Brd4 in nuclear foci in the absence or presence of the origin containing plasmid, at least five cells were digitally reconstructed from each condition by deconvolution of high resolution 3D stacks of confocal images. The replication foci were demarcated using the Surpass module of Imaris software (Bitplane) to define volumes expressing high levels of the E1 protein. These foci are shown in green on the image in the left panel. The proportion of E1, E2 and Brd4 in the E1 foci (levels in E1 foci volume/levels in nuclear volume) is represented in the graph in the middle. The graph on the right shows the enrichment of each protein in the E1 foci relative to the volume of the nucleus (levels of each protein multiplied by volume of E1 foci/volume of nucleus). Average values obtained from at least five reconstructed nuclei are shown and the error bars represent the standard deviation.

In our previous study we had shown that addition of the HPV genome, or a plasmid containing the origin, caused the foci to greatly increase in size, indicative of active viral genome replication [4]. To further determine the requirement for the Brd4 protein in the presence of an actively replicating genome, we examined its localization in the presence of HPV16 E1 and E2 proteins and a co-transfected viral origin (p16ori) or HPV16 genome. As shown in Figure 3, Brd4 speckles were no longer highly enriched within the E1–E2 foci in the presence of a replicating genome or origin. While Brd4 was still enriched in some foci, it also showed adjacent or perimeter staining in others. When the foci became really large, Brd4 was often completely absent. In general, Brd4 was present in the smaller E1/E2 foci and was displaced or absent as the foci became larger. Figure 3A shows examples of foci with and without the origin. Notably, in the absence of the origin, E1 and Brd4 are greatly enriched in the nuclear foci while E2 only partially localizes here (though E2 is essential for the development of the foci). However, in the presence of the origin there is much more complete localization of E2 within the large replication foci. This observation is supported by the quantitation reported in the graphs shown in Figure 3B. In the absence of the origin, approximately 50–60% of nuclear Brd4 is recruited to the E1 foci but in the presence of the origin this drops to just over 20%, although there is no significant change in the overall levels of Brd4. Conversely, only about 25% of the E2 protein is present in the E1 foci in the absence of the origin, but this increases to approximately 40% in the presence of the origin. Thus, the presence of the origin plasmid stabilizes the association of E1 and E2, but decreases the association of Brd4 with E1/E2 nuclear foci. These experiments suggest that Brd4 is required at an early stage in the development of the replication foci, but that it is displaced or dispersed as the genomes or origin plasmids replicate and the replication foci grow in size.

### Brd4 is displaced in the presence of the replication origin as foci increase in size

**Figure 4:**
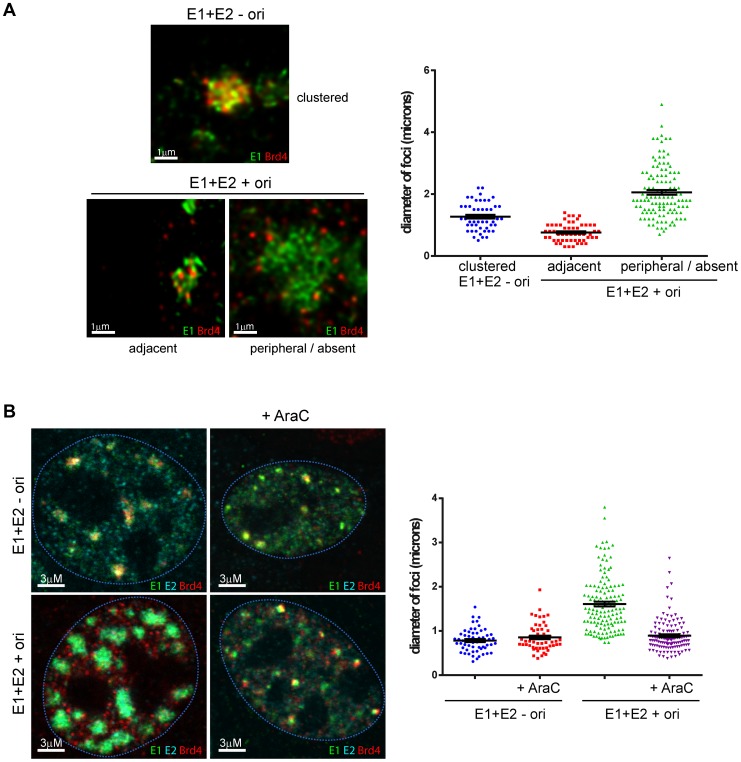
In the presence of a replicating viral origin, the size of E1/E2 foci increases and Brd4 is displaced. **A.** Human keratinocytes were cotransfected with expression vectors for HPV16 E1 and E2 in the presence of a plasmid containing the minimal HPV16 replication origin, p16ori (+ ori) or a control plasmid without the origin, pKS (− ori). The E1 and Brd4 proteins were detected by immunofluorescence. Representative foci from each condition are shown. The diameter of E1 foci was measured using Bitplane Imaris. Error bars represent the standard error of the mean. **B.** Human keratinocytes were cotransfected with expression vectors for HPV16 E1 and E2 in the presence of a plasmid containing the minimal HPV16 replication origin, p16ori (+ ori) or a control plasmid without the origin, pKS (− ori). E1 and E2 expression was induced four hours before fixation in the presence of 25 µg/ml AraC and E1, E2 and Brd4 proteins were detected by immunofluorescence. Representative cells from each condition are shown. The diameter of E1 foci was measured using Bitplane Imaris. Error bars represent the standard error of the mean.

To further confirm and quantitate the observation that the E1/E2 foci grow in size in the presence of a plasmid that contains the viral origin, and that this correlates with displacement of Brd4, the diameter of individual foci formed in the absence and presence of the viral origin containing plasmid was measured (Figure 4A). Foci formed in cells co-transfected with the origin containing plasmid were further divided into two phenotypes: foci in which Brd4 was clearly adjacent or overlapping and foci where Brd4 was either peripheral or absent. As shown in Figure 4, E1/E2 foci were approximately 1–2 µm in diameter in the absence of the origin, and showed great enrichment for the Brd4 protein. In the presence of the origin, foci that were enriched for Brd4 tended to be small (1 µm or less), and those that had only peripheral Brd4 speckles or were deficient in Brd4 tended to be larger (average diameter >2 µm). Taken together, these findings imply that Brd4 is associated with small, nascent replication foci, but that as the viral origin replicates the foci grow in size and displace Brd4.

### DNA replication inhibitors abrogate the expansion of foci in the presence of the replication origin

To further show that the expansion of the E1/E2 foci in the presence of the origin was due to replication, cells were treated with cytosine arabinoside (AraC), a DNA synthesis inhibitor, after cotransfection of the E1/E2 and origin containing plasmids. As shown in Figure 4B, AraC had little effect on the size of the E1/E2 foci in the absence of the origin, but in the presence of the origin it inhibited the ori-dependent growth in size of the foci. This provides further evidence that the expansion of the E1/E2/ori foci is due to DNA replication of the origin containing replicon.

### HPV origin containing plasmids replicate to high levels in E1/E2 nuclear foci

**Figure 5:**
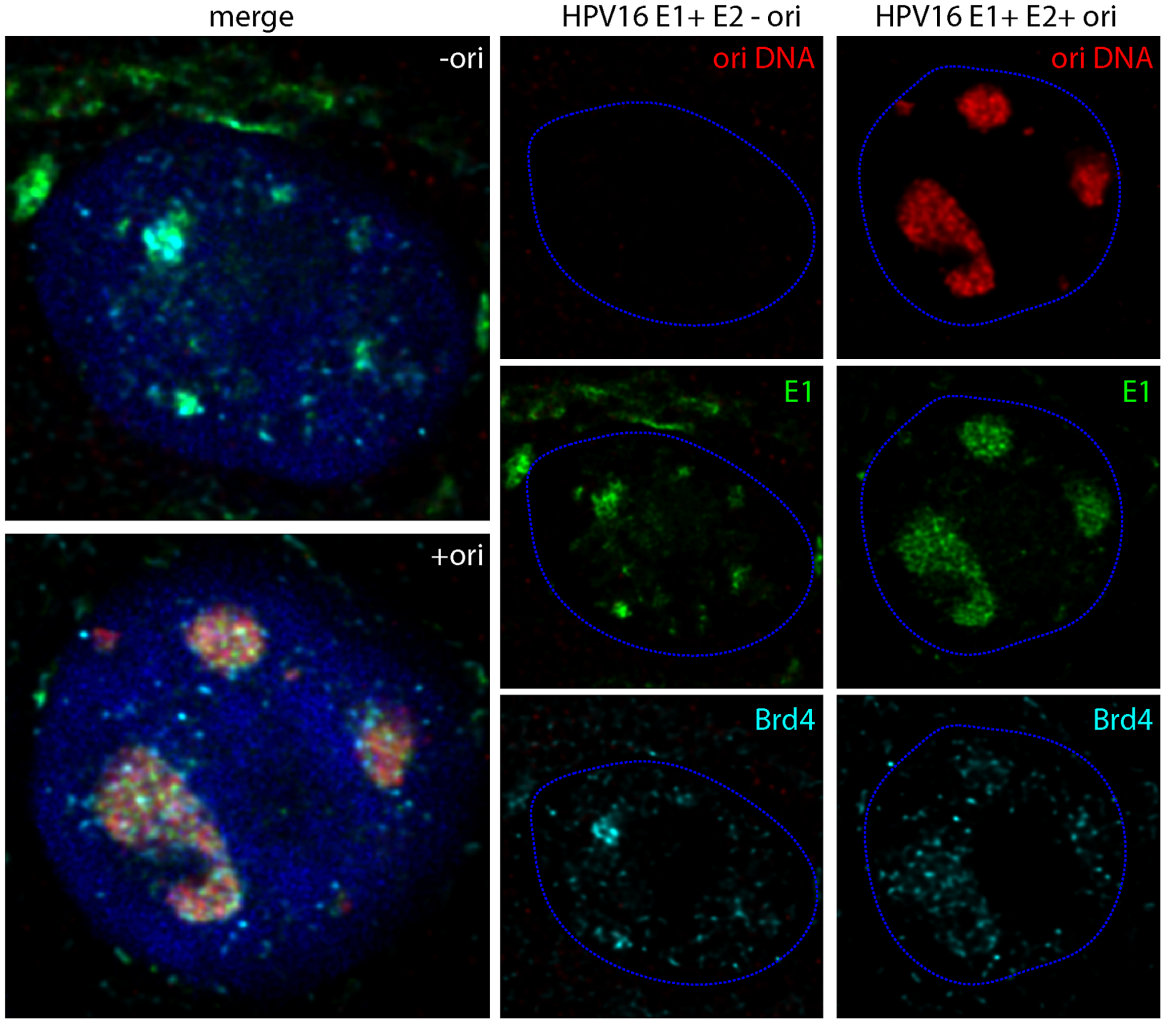
HPV origin containing plasmids replicate to high levels in E1/E2 nuclear foci. Human keratinocytes were cotransfected with expression vectors for HPV16 E1 and E2 in the presence of a plasmid containing the minimal HPV16 replication origin, p16ori (+ ori) or a control plasmid without the origin, pKS (− ori). The E1 and Brd4 proteins were detected by immunofluorescence, and following a brief fixation, origin containing DNA was detected by FISH. The images shown are optical slices from cells that were digitally reconstructed by deconvolution of high resolution 3D stacks of confocal images. E1 is shown in green, Brd4 in cyan, and origin DNA in red. The dashed blue lines represent the perimeter of the nuclei (identified by DAPI staining, not shown).

To further ensure that the expansion of the E1/E2 foci observed in the presence of the origin plasmid was actually due to viral DNA replication, we carried out combined IF-FISH for E1 and Brd4 and for origin containing DNA. Keratinocytes transfected with E1 and E2, and either a co-transfected viral origin containing plasmid (p16ori) or control plasmid (pKS) were analyzed for E1, Brd4 and viral origin DNA. As shown in Figure 5, the large foci resulting from cotransfection with the origin plasmid contained high levels of viral DNA, while no signal was observed in cells transfected with the control plasmid. Both the E1 protein and HPV origin plasmid completely filled the large foci in an overlapping localization. Small speckles of Brd4 were located on the periphery of the foci, as shown in Figure 5, or were absent. Careful observation of 3D nuclei reconstructed from deconvolved stacks of confocal images showed that the small speckles of E1 and origin DNA did not completely overlap but were clustered together within the foci. Similar results were obtained when recircularized HPV16 genome was cotransfected with the E1 and E2 expression plasmids. Thus, the expansion of the E1/E2 foci observed in the presence of a viral origin is associated with viral DNA replication.

### Brd4 surrounds replication foci in differentiating cells that are amplifying the viral genome

The experiments above were carried out in keratinocytes transiently expressing only the E1 and E2 proteins. To analyze the involvement of Brd4 in replication foci in cells naturally infected with HPV genomes, we analyzed the localization of Brd4 in CIN-612 9E cells. These cells were established from a cervical CIN lesion, harbor about 500 copies of the HPV31 genome and can be readily induced to differentiate using calcium, methyl cellulose or organotypic raft culture [9], [21]–[23]. HPV replication foci can be detected in these cells by staining for γH2AX (or other markers of the DNA damage response), and in many cells these foci are observed to increase in size upon differentiation and to contain amplifying viral DNA [9].

**Figure 6:**
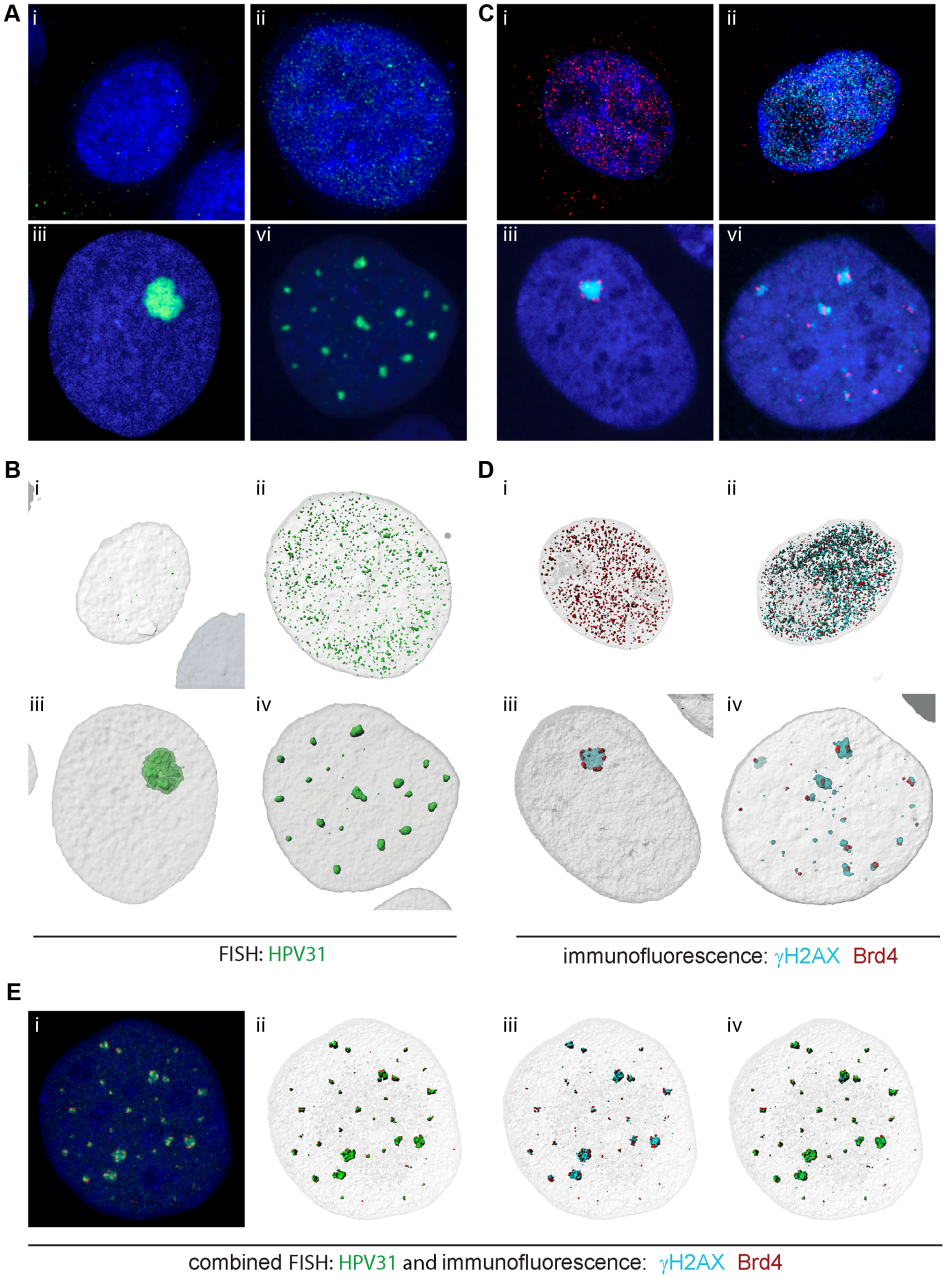
Brd4 surrounds replication foci in differentiating cells harboring HPV31 genomes. **A and B** Replication foci were detected by FISH for HPV31 DNA in uninfected keratinocytes (i), and undifferentiated (ii) and differentiated (iii and iv) CIN-612 9E cells. **C and D** Replication foci were detected by immunofluorescence for γH2AX (cyan) and were also stained for Brd4 (red) DNA in uninfected keratinocytes (i), and undifferentiated (ii) and differentiated (iii and iv) CIN-612 9E cells. **A, B, C, D**. 3D image stacks were deconvolved and digitally reconstructed using Huygens Essential software. Volume images are shown in A and C and high resolution surface renderings of all objects were generated by Bitplane Imaris software and are shown in B and D. **E** The γH2AX and Brd4 proteins were detected by immunofluorescence in differentiated CIN-612 9E cells., and following a brief fixation, viral DNA was detected by FISH. Volume images are shown in i and high resolution surface renderings of all objects were generated by Bitplane Imaris software and are shown in ii, iii and iv. γH2AX is shown in cyan, Brd4 in red, and HPV31 DNA in green.

When analyzed by FISH, undifferentiated 9E cells contain either undetectable (data not shown) or many tiny speckles of HPV31 DNA (Figure 6A and B). Differentiated 9E cells contain both small (Figure 6Aiv and 6Biv) and large (Figure 6A iii and 6B iii) replication foci. Co-staining of these cells with antibodies to γH2AX and Brd4 showed a similar pattern (Figure 6C and D). Uninfected keratinocytes and undifferentiated 9E cells showed tiny speckles of Brd4 distributed throughout the nucleus (Figure 6Ci and 6Di and 6Cii and 6Dii). 9E cells also contained many small speckles of γH2AX that resembled the HPV31 signal in Figure 6Aii. In differentiated cells the γH2AX pattern resembled the small and large replication foci detected by FISH. The overall Brd4 signal was greatly reduced in differentiated cells; Brd4 is a proliferation-associated factor and levels diminish upon differentiation of the epithelium (data not shown). However, small Brd4 speckles were observed adjacent to the small γH2AX replication foci (Figure 6Civ and 6Div) and surrounding the large foci (in about 50% of cells; Figure 6Ciii and 6Diii). In Figure 6E, we show by combined IF-FISH, that the γH2AX is intermingled in the foci with HPV31 DNA, and the resulting foci are covered with speckles of Brd4. This result is consistent with the experiments shown in Figure 3, when addition of a replication competent genome or replication origin results in displacement of Brd4 from replication foci.

### HPV16 E1/E2 and HPV31 9E foci contain markers of homologous recombination

We have previously proposed that papillomaviruses might take advantage of the DNA damage response in differentiated cells to recruit DNA repair machinery to enhance differentiation-dependent genome amplification in cells that have exited the cell cycle [4], [8]. Furthermore, Gillespie et al. have noted that markers of homologous recombination are localized with HPV31 foci in 9E cells [10]. Therefore, we also examined the different types of foci for the presence of Rad51, a classical marker of homologous recombination.

**Figure 7:**
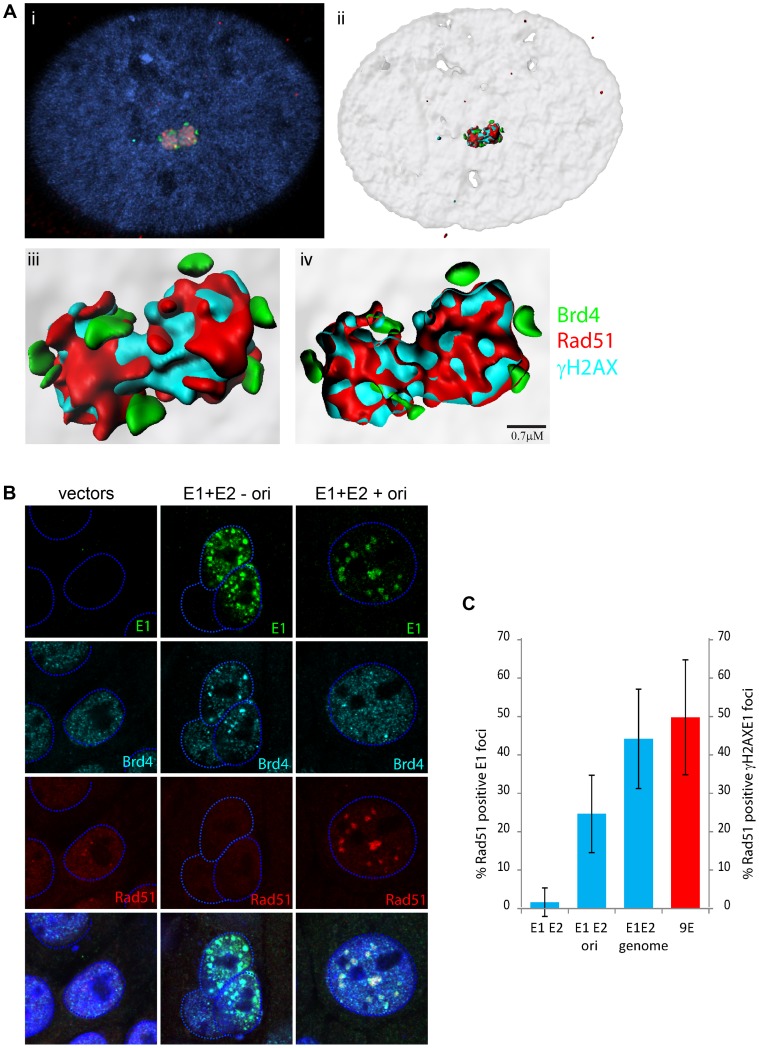
Homologous recombination marker, Rad51, colocalizes with HPV replication foci. **A.** (i) Nuclear foci were generated by differentiation of CIN612 9E cells for five days. Cells were stained with antibodies against Brd4 (rabbit mcb2), Rad51 (mouse) and γH2AX (mouse). Isotype specific secondary antibodies were used to distinguish between the mouse antibodies. Cellular DNA is counterstained with DAPI (blue in merged panels). 3D image stacks were deconvolved using Huygens Essential software.  (ii) High resolution surface renderings of all objects generated by Bitplane Imaris software. (iii) High resolution surface rendering of the replication foci shown in (ii). (iv) Cross-section through the replication foci shown in (i, ii, iii). Rad51 and γH2AX are localized within the foci and are surrounded by small speckles of Brd4. **B.** Nuclear foci were generated by transfection of HPV16 E1 and E2 expression vectors (with and without p16ori). Cells were stained with antibodies against E1, Brd4 (rabbit mcb2) and Rad51 (mouse). The dashed blue lines represent the perimeter of the nuclei (identified by DAPI staining, not shown). **C.** The percentage of HPV16 E1/E2 foci containing Rad51, obtained from experiments as described in B, is represented by the blue bars. The percentage of γH2AX foci containing Rad51 in differentiated CIN612 9E cells (as described in A) is represented by the red bar. Error bars represent standard deviation.

Many viruses switch to a recombination-dependent replication mode at late stages of replication (reviewed in [8]), and this often involves a recombinase such as Rad51 which promotes strand invasion and homologous pairing. As shown in Figure 7, the HPV31 9E foci costain with an antibody that recognizes Rad51. Not all γH2AX foci are positive for Rad51 (about 50%, see Figure 7C), but in general the larger, presumably more mature foci stain with Rad51 at the center of the foci. High resolution analysis and surface rendering show that Rad51 and γH2AX localize in a mutually exclusive pattern in the foci (Figure 7A), and analysis of many cells shows that Rad51 forms at the core of the foci while γH2AX forms a cloud around the foci. Brd4 is located on the periphery of the foci in a satellite pattern in 9E cells.

HFK cells expressing the E1 and E2 proteins were also examined for Rad51 localization to the E1/E2 or E1/E2/p16ori foci. As shown in Figure 7B, the foci formed in the presence of E1, E2 and an origin containing plasmid also contained Rad51 in about 25% cells. However, this was not observed in the absence of the origin containing plasmid. Cotransfection with a recircularized genome, instead of the origin plasmid, raised this to over 40%. Furthermore, Rad51 staining in the foci could be detected in both undifferentiated and differentiated keratinocytes (data not shown). Therefore, the E1 and E2 proteins alone are capable of inducing replication foci that can recruit proteins involved in homologous recombination, but this depends on the presence of replicating viral DNA.

### E1–E2 foci are associated with similar chromatin modifications to those formed in differentiated HPV containing cells and contain markers of homologous recombination

**Figure 8:**
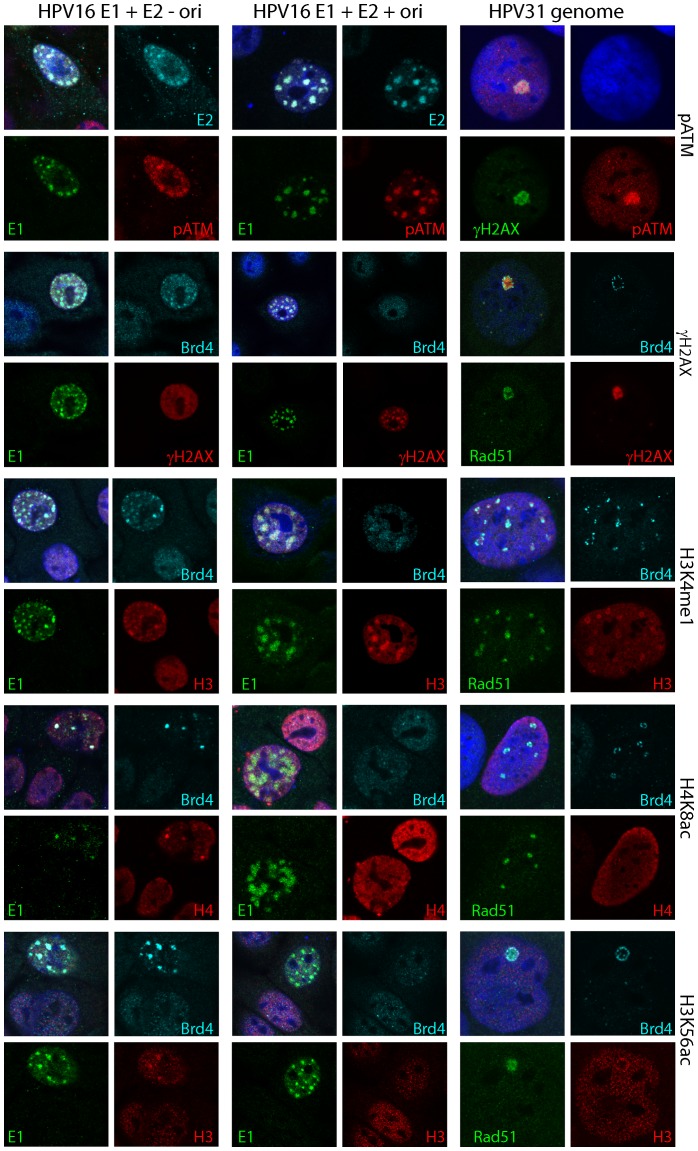
Comparison of the characteristics of foci formed by expression of E1 and E2 proteins and naturally occurring replication foci in differentiated HPV containing cells. HFKs were transiently transfected with HPV16 E1 and E2 expression plasmids and the control plasmid pKS (left column), or with HPV16 E1 and E2 expression plasmids and the replication origin plasmid p16ori (middle column). HPV31 genome containing 9E cells (right column) were differentiated for five days as described in Methods. Cells were stained for E1, E2, Brd4, H3K4me1, H4K8ac and H3K56ac, Rad51, pATM and γH2AX, as indicated. The rabbit Brd4 mcb2 antiserum is used in most panels, except for H3K4me1, H4K8ac and H3K56ac staining in HPV31 9E cells (right column), where a mouse Brd4 antibody 8H2 was used. A goat anti-Rad51 antibody was also used in these panels. A mouse Rad51 antibody was used to stain HPV31 9E cells, along with Brd4 (rabbit mcb2) and γH2AX (mouse). Isotype specific secondary antibodies were used to distinguish between the mouse antibodies. Cellular DNA is counterstained with DAPI (blue in merged panels).

Nuclear foci formed by the E1 and E2 proteins, or by induction of differentiation in HPV containing cell lines, recruit markers of DNA synthesis and the DNA damage response. Nevertheless, there is debate as to whether these foci are similar and comparable. Since we are investigating the role of Brd4, a chromatin adaptor protein, in the formation of these foci, we analyzed the presence of histone modifications in and around the nuclear foci. As shown in Figure 8, we analyzed these markers in foci generated by expression of HPV16 E1 and E2, E1 and E2 plus an origin containing plasmid, or HPV31 foci formed in differentiated 9E cells. We examined pATM, γH2AX, H3K4me1, H4K8ac and H3K56ac.

As shown previously, HPV31 9E foci and E1–E2 foci contain ATM phosphorylated on serine 1981 [4], [10]. As shown in Figure 8, pATM is also retained in E1–E2 foci actively replicating an origin, so this is a consistent marker for all three types of foci. Cells containing these three types of foci are also γH2AX positive [4]–[6]. However, in the absence of the origin, while localized to the foci, γH2AX is also found in a diffuse pattern throughout the nucleus. In the presence of the origin, γH2AX is much more often localized more exclusively with the E1 and E2 replication foci. A similar situation is found in the HPV31 CIN612 9E cells, where γH2AX staining overlaps with viral DNA in the middle-sized and large replication foci ([24], Figure 6 and data not shown).

H4K8ac is a binding target of the Brd4 bromodomains [25] and it strongly overlaps with the Brd4 positive E1/E2 foci. In the E1/E2/ori foci, H4K8ac was only partially localized with the foci in a manner similar to that of Brd4. Likewise, in 9E cells H4K8ac formed a satellite pattern similar to that of Brd4 in some, but not all cells. Similarily, H3K56ac stained only the foci formed by the E1 and E2 proteins but not those containing a replicating origin or the HPV31 9E replication foci.

In contrast, H3K4me1, a marker for active chromatin and enhancer regions [26] co-localized with all three types of foci. In 9E cells it stained the replication foci but was not necessarily coincident with the Brd4 satellite staining pattern. It will be informative to determine whether viral chromatin contains H3K4me1 and whether this is a signature of amplified DNA. Therefore, these chromatin markers demonstrate a shift in histone modifications as the E1/E2 foci switch to true E1/E2/origin replication foci. Moreover, the latter foci have very similar characteristics to those of HPV31 CIN612 9E cells.

As summarized in Table 1, in the absence of a viral replicon, the E1/E2 foci are strongly co-localized with E1, Brd4 and pATM together with H3K4me1, H4K8ac and H3K56ac markers. E2 and γH2AX, while partially localized to the foci are also often throughout the cell. In E1/E2 foci in the presence of a replicon, or in HPV31 CIN612 9E foci, E1 and E2 (in the transfected cells), pATM and γH2AX colocalize strongly in large replication foci. These foci also stain with H3K4me1 (in a γH2AX like localization), but H3K56ac does not colocalize. H4K8ac is either absent, dispersed or in a peripheral pattern similar to that observed with Brd4. We believe that these changes represent a switch to full-blown replication foci. As discussed above, the location of Brd4 and acetylated histones could correspond to cellular chromatin displaced by the expanding, replication competent foci.

**Table 1:** Properties of replication foci.

Property	Details	HPV16 E1–E2 (in HFK)	HPV16 E1–E2 plus origin DNA (in HFK)	HPV31 genomes (in CIN612 9E)
**Histone modifications**	Brd4	overlap	Partial overlap/adjacent	adjacent
	H4K8ac	overlap	Partial overlap/adjacent	adjacent
	H3K56ac	overlap	no colocalization	no colocalization
	BET inhibitors	disrupts foci	foci resistant	foci resistant^1^
	TSA	disrupts foci	foci resistant	foci resistant^1^
	H3K4me1	overlap	overlap	overlap
**DNA damage response**	pATM, γH2AX, pNBS1	colocalize	colocalize	colocalize
	KU-55933 and caffeine	foci resistant	foci resistant	foci partially resistant^1^
**Homologous Recombination**	Rad51	negative	colocalize	colocalize
**HPV Genetics**	E2 R37A I73A; E2 R37K; E2 I73L	defective for focus formation	competent for focus formation	E2 R37K and I73L focus formation similar to wild type HPV31

1 differentiated 9E cells contain a very heterogeneous mix of cell and foci phenotypes and it is difficult to determine more subtle effects on the distribution of different foci types using this method.

### Disruption of Brd4 function or expression abolishes the formation of E1–E2 foci but not E1–E2 foci actively replicating viral DNA

**Figure 9:**
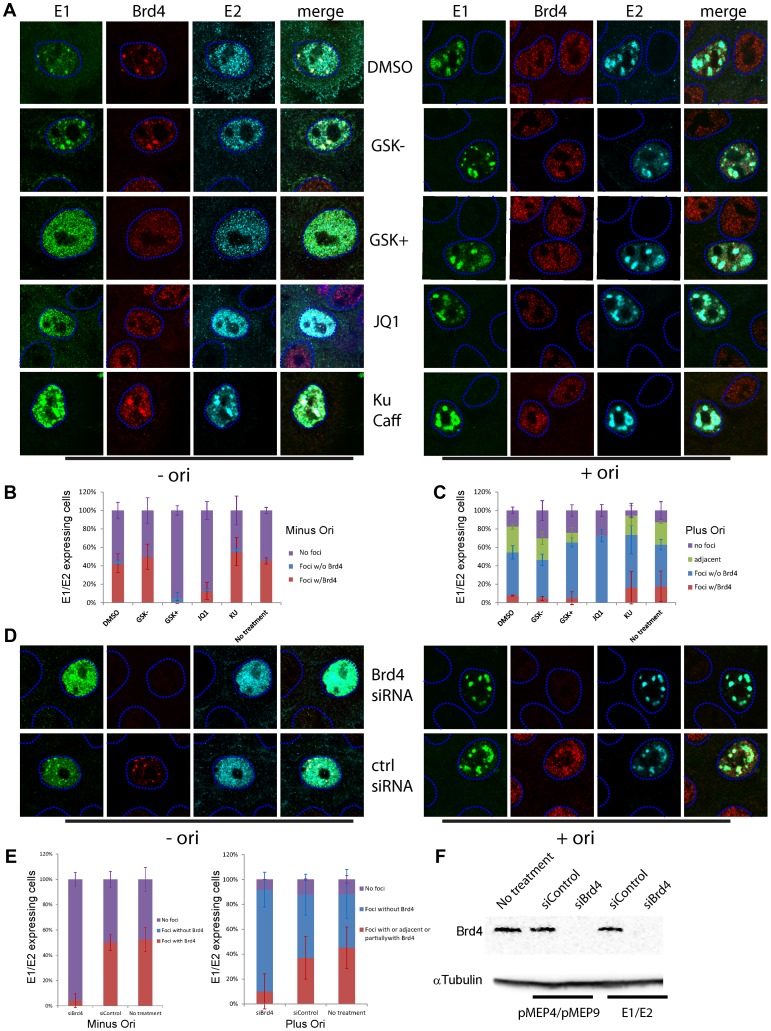
Brd4 is required for the formation of E1–E2 replication foci but viral genome replication obviates this need. **A.** Effect of BET inhibition of Brd4 chromatin binding on E1–E2 foci. Keratinocytes were transfected with HPV16 E1 and E2 and treated with DMSO (solvent), 2 µM GSK525762A+ (a BET inhibitor) and 2 µM GSK525762A− (an inactive enantiomer of GSK525762A+), 0.5 µM JQ1 or a mixture of 10 mM KU-55933 and 2.5 mM caffeine. Replication foci were detected by immunofluorescence for E1 (green), Brd4 (red) and E2 (cyan). **B.** The percentage of E1/E2 expressing cells with nuclear E1/E2 containing foci with or without Brd4 after treatment with BET inhibitors. **C.** The percentage of E1/E2 expressing cells with nuclear E1/E2 containing foci with or without Brd4 in the presence of an origin containing replicon (p16ori) after treatment with BET inhibitors. **D.** HFKs were treated with either Brd4 siRNA or control siRNA for 24 hours before transfection with HPV16 E1 and E2 expression plasmids. Replication foci were detected by immunofluorescence for E1 (green), E2 (cyan) and Brd4 (red). The dashed blue lines represent the perimeter of the nuclei (identified by DAPI staining, not shown). **E.** The percentage of E1/E2 expressing cells with nuclear E1/E2 containing foci with or without Brd4 after treatment with siRNA to Brd4 in the presence or absence of an origin containing replicon after treatment with siRNA to Brd4. **F.** Western blot showing reduction of Brd4 expression upon siBrd4 treatment.

Small molecules that specifically inhibit the binding of Brd4 bromodomains to acetylated histone tails have recently been described [27], [28]. The BET family inhibitors, JQ1 and GSK525762A+ are mimics of the acetylated lysine residues of histone tails and GSK525762A− is an inactive enantiomer of GSK525762A+ and serves as a negative control. These reagents enabled us to determine the requirement for Brd4 in the formation of HPV replication foci. The molecules JQ1, GSK525762A− and GSK525762A+ were chemically synthesized and used to treat cells for 24 hours before E1 and E2 expression. As shown in Figure 9A and 9B, JQ1 and GSK525762A+ abolished the formation of nuclear foci in cells expressing E1 and E2, while GSK525762A− or the solvent DMSO had no effect. Therefore, the ability of Brd4 to bind to acetylated chromatin is required for E1/E2 foci. However, addition of the origin plasmid abrogated the requirement for Brd4 chromatin binding. As shown in Figure 9A and 9C, JQ1 and GSK525762A+ no longer disrupted the formation of E1–E2 replication foci, and the foci that formed showed little if any, recruitment of Brd4.

We also treated differentiated HPV31 containing 9E cells with the BET inhibitors and examined the formation of replication foci in these cells (data not shown). While the BET inhibitors greatly disrupted the satellite localization of Brd4 around or adjacent to replication foci, it was difficult to conclude that there was a substantial effect on the formation of small or large replication foci. In addition, it was very difficult to accurately quantitate more subtle effects in these cells because of the very heterogeneous nature of the differentiated cells and foci. And finally, we find that the BET inhibitors inhibit keratinocyte differentiation (discussed below), which further complicates the analysis in differentiated cells. Further studies are required in a more homogeneous population of cells to firmly conclude whether the BET inhibitors modulate different stages of replication foci.

In theory, histone deacetylase (HDAC) inhibitors such as trichostatin A (TSA) should increase global acetylation and promote binding of bromodomain containing proteins to chromatin. In practice, binding of these proteins to specific genomic regions is lost because of competition with high levels of acetylated chromatin throughout the nucleus [29], [30]. Accordingly, TSA treatment disrupts the formation of E1–E2-Brd4 foci in keratinocytes (data not shown). However, TSA did not disrupt replication foci formed with E1, E2 and the p16ori replicon, or replication foci in differentiated 9E cells.

Notably the formation and growth of E1–E2 foci was not disrupted by the presence of KU-55933 and caffeine, inhibitors of the ATM and ATR DNA damage response pathways (Figure 9 A–C). In fact, it was easier to find foci expressing the E1 and E2 proteins since the inhibition of the DNA damage response prevented the previously described growth arrest and cell death resulting from E1 and E2 expression [4], [6]. Preliminary data indicate that KU-55933 and caffeine do not eliminate viral DNA replication within the foci (data not shown), consistent with the finding that it is only differentiation-dependent amplificational replication that is enhanced by functional ATM pathway [9].

To further determine whether the presence of the Brd4 protein was essential for formation of the E1–E2 foci, its expression was downregulated using siRNA. As shown in Figure 9D and 9F, Brd4 expression could be drastically reduced, as observed by immunoblotting and immunofluorescence. When Brd4 expression was abolished, expression of E1 and E2 no longer gave rise to nuclear foci in the absence of the origin plasmid; the number of cells containing nuclear foci in the presence and absence of Brd4 are shown in Figure 9E. However, consistent with the results obtained with the BET inhibitors, inhibition of Brd4 expression or chromatin binding was not detrimental to the formation of E1 and E2 foci in the presence of a viral replicon. Cells treated with Brd4 siRNA or BET inhibitors could still form E1/E2 containing replication foci (Figure 9E). Therefore, Brd4 is not essential for the formation and growth of replication foci in cells containing moderate amounts of a viral replicon; when the viral genome has begun to actively replicate, and recruit the necessary factors for DNA synthesis, Brd4 is no longer necessary. Treatment of HPV31 containing 9E cells with Brd4 siRNA gave a similar result to treatment with the BET inhibitors: there did not appear to be a substantial effect of reducing Brd4 expression on the formation of replication foci (data not shown). However, this experiment suffered from the same complication that the heterogeneous nature of the differentiated cells and replication foci made it difficult to elucidate more subtle effects on the formation of HPV replication foci.

A seemingly straightforward experiment to assess the role of Brd4 on differentiation-dependent HPV replication would be to downregulate Brd4 expression or disrupt Brd4 chromatin binding in cells that are actively replicating the genome and assess levels of viral DNA. However, disruption of Brd4 expression or function is detrimental to cell growth, thus making the results difficult to interpret. Moreover, the E2-Brd4 interaction is important for viral transcription [31], [32], which could have indirect effects on viral replication. Nevertheless, because the HPV31 9E cells are differentiated to induce vegetative viral replication it seemed worth assessing the role of the BET inhibitors in this process. JQ1 and GSK525762A+ were tested for their ability to inhibit vegetative amplification in keratinocytes containing replicating HPV31 genomes. Unfortunately, these small molecules also inhibited differentiation of HPV-containing keratinocytes (data not shown) and so we were unable to use them for this purpose.

### Functions of E1 and E2 required for localization of Brd4 to replication foci in the presence and absence of an HPV replicon

**Figure 10:**
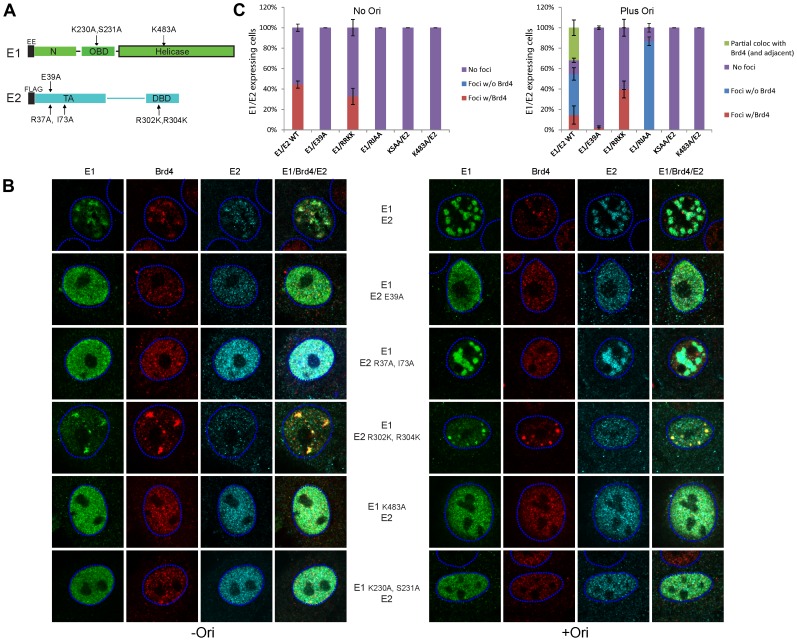
Analysis of the E1–E2 functions required for localization of Brd4 to nuclear E1–E2 foci in the presence and absence of viral origin containing DNA. **A.** Representation of mutations that specifically inactivate E1 and E2 functions. E1: K230A, S231A (defective in specific DNA binding); K483A (ATPase deficient). E2: E39A (deficient in interaction with E1); R37A, I73A (deficient in transcriptional regulation and Brd4 binding); R302K, R304K (DNA binding defective). **B.** Wild-type or mutated HPV16 E1 and E2 expression plasmids were transiently transfected into keratinoctytes and cells were stained by immunofluorescence for E1 (green), E2 (cyan) and Brd4 (red). The dashed blue lines represent the perimeter of the nuclei (identified by DAPI staining, not shown). The cells in the left panel were also transfected with a control plasmid (pKS) and those on the right with a minimal origin containing plasmid (p16ori). Fluorescent signals were collected at unsaturated levels, according to viral protein expression, and were later increased to enhance figure brightness. **C.** Cells expressing E1 and E2 in the presence or absence of origin containing DNA were counted and scored for the presence of foci that co-localized with Brd4.

Because of the complications described above in disrupting Brd4 expression and function, we took a genetic approach to further analyze the role of Brd4 in viral replication foci. We had previously analyzed the ability of HPV16 E1 and E2 proteins with mutations in very specific functions for their ability to form foci and to induce the ATM/ATR response [4]. E1 and E2 proteins with the same mutations were tested for their ability to colocalize with Brd4 (Figure 10). In the absence of the origin, the mutated E1 proteins and the E2 protein with the E1 interaction mutation were not able to form foci and so we could not examine colocalization with Brd4. Likewise, the E2 protein mutated in R37A/I73A could not form foci. Only the E2 protein mutated in the specific DNA binding function (R302K/R304K) was able to form foci and these foci overlapped with Brd4 like the wild type proteins. Thus, the DNA binding function of E2 is not required for the formation of nuclear foci or for the localization of Brd4 to these foci.

Consistent with other experiments in this study, the requirements for the formation of the E1–E2 foci were somewhat different in the presence of an HPV-derived replicon or genome. The mutated E1 and E2 proteins were tested for their ability to form foci in the presence of the viral genome or an origin containing plasmid. As before, a functional E1 protein was required for the formation of nuclear foci, but the E2 requirements were different. Somewhat surprisingly, when a replication competent genome or origin was present, E2 R37A/I73A could form foci with E1. These foci were large and prominent (similar to the wildtype foci actively replicating genomes) but they did not localize with Brd4. On the other hand, a specific DNA binding defective E2 protein could still form foci with E1 that co-localized with Brd4, but these foci did not increase in size, or change or displace Brd4 when cotransfected with an origin plasmid. This is consistent with the inability of this protein to bind to and promote replication of the origin. Thus, in the presence of the origin, the foci greatly increase in size and Brd4 is not required for this phenomenon. The localization patterns of Brd4 observed with wildtype E1–E2 foci is consistent with the theory that Brd4 is displaced to the periphery of the foci as high level replication gets underway.

### HPV31 genomes mutated in E2 residues important for Brd4 interaction can stably replicate in keratinocytes

**Figure 11:**
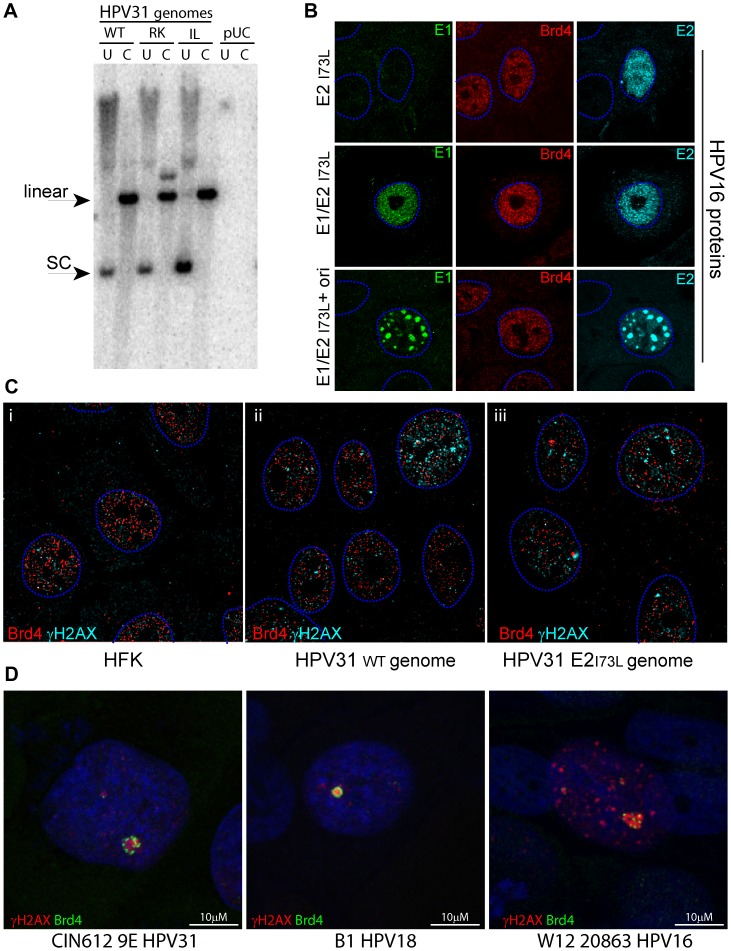
Replication and nuclear localization of HPV31 genomes containing mutations in E2 that disrupt Brd4 binding. **A.** HFK cells were transfected with recircularized HPV31 genomes as described in Methods. The resulting drug-resistant colonies were pooled, cultured for eight passes and analyzed by Southern blot analysis. WT: wild type genome; RK: HPV31 E2 R37K; IL: HPV31 E2 I73L; pUC: control plasmid transfected cells. Total cellular DNA was either uncut (U) or digested with a restriction enzyme that cleaved the HPV31 genome once (C). The linear and supercoiled forms of the HPV31 genomes are indicated. **B.** An expression plasmid encoding HPV16 E2 with an I73L substitution mutation was transiently transfected into keratinoctytes with either control empty vectors, pMEP9 and pKS (top row); or HPV16 E2 I73L and wildtype E1 expression plasmids and control empty vector, pKS (middle row); or HPV16 E2 I73L, HPV16 E1 and p16ori plasmids (bottom row). Cells were stained by immunofluorescence for E1 (green), E2 (cyan) and Brd4 (red). The dashed blue lines represent the perimeter of the nuclei (identified by DAPI staining, not shown). **C.** Immunofluorescence of differentiated normal HFKs and the HFKs described in A (immortalized with HPV31 wildtype and HPV31 E2 I73L genomes. Cells were differentiated with calcium for five days and stained for viral replication foci with γH2AX (red) and Brd4 (green). The dashed blue lines represent the perimeter of the nuclei (identified by DAPI staining, not shown). 3D image stacks were reconstructed by deconvolution using Huygens Essential software, and a single slice is shown. The scale bar represents 5 µm. **D.** Immunofluorescence of differentiated CIN-612 9E cells, W12 20863 cells, and B1 (a laboratory generated HPV18 cell line). Cells were differentiated with calcium for five days and stained for viral replication foci with γH2AX (red) and 2290, an antibody that recognizes all forms of Brd4 (green). Nuclei were identified by DAPI staining. The scale bar represents 10 µm.

An important study by Stubenrauch and colleagues showed that HPV31 genomes with conservative substitutions in residues important for E2-mediated transcriptional regulation (R37K and I73L) were able to replicate in keratinocytes and induce late viral replication and transcription [33]. These mutations were later found to disrupt the interaction of E2 with the Brd4 protein [12], [15]. Thus, we obtained these genomes to further investigate the role of Brd4 in viral replication foci. As described previously, the mutated HPV31 genomes could efficiently replicate extrachromosomally in keratinocytes (see Figure 11A).

We tested the ability of the equivalent HPV16 E2 R37K and I73L proteins to bind Brd4 in vitro and found that they were very defective (<20% wildtype E2 binding; data not shown). We also tested the ability of the HPV16 E2 R37K and I73L proteins to form nuclear foci in the presence or absence of the viral origin plasmid. As we had found for HPV16 E2 R37A/I73A, these proteins were defective in their ability to form foci in the absence of the origin, but could form large foci in the presence of the origin. Figure 11B shows examples of these foci in cells expressing HPV16 E1 and E2 I73L. Therefore, the conservative I73L mutation gives the same phenotype as the R37A/I73A mutation.

Stable keratinocyte cell lines were generated with the HPV31 genomes with E2 R37K and I73L mutations (shown in Figure 11A) and we examined the relative localization of γH2AX and Brd4 in these cells. However, it was difficult to observe the strong Brd4 satellite pattern that is so apparent in HPV31 9E cells. Figure 11C shows the localization of γH2AX and Brd4 in deconvolved 3D stacks of images from representative cells. These cells contain many small speckles of γH2AX and Brd4; a few of the Brd4 speckles are in close association with γH2AX foci, but it is difficult to conclude that this association is specific and that there is a difference between the wild type (Figure 11C ii) and mutated (Figure 11C iii) genomes. Therefore, we conclude that either there is no specific association between Brd4 and the γH2AX foci in these cells, or else the association between Brd4 and the foci is much less distinctive than observed in 9E cells and does not involve the key E2 residues, R37 and I73.

Because of the difficulties in observing the satellite Brd4 pattern in the HPV31 cell lines, we examined the localization of Brd4 in two additional cell lines that contain replicating HPV genomes. As shown in Figure 11D, Brd4 was often found in a satellite pattern around the γH2AX replication foci in differentiating cells containing HPV16 (the 20863 clone of W12 cells, a clinical isolate which maintains the viral genome as a high copy number episome [34]) or a laboratory generated keratinocyte line containing HPV18 viral genomes. Notably, Brd4 was much more readily detected in this location with an antibody against the N-terminal half of the Brd4 protein, which can detect all isoforms of Brd4. As discussed below, we believe that the association between γH2AX replication foci and Brd4 is observed most prominently in genetically unstable cells.

## Discussion

### Summary

In this study we analyze the role of the chromatin adaptor protein Brd4 in HPV replication. We show that Brd4 is specifically localized to nuclear foci formed by expression of the E1 and E2 replication proteins in keratinocytes, and is required for the formation of these foci. However when the foci begin to actively replicate viral DNA and grow, either with time post-transfection of an origin containing replicon or after amplification of viral genomes by differentiation of host cells, Brd4 is dispersed or observed localized adjacent to these foci and is no longer required for their formation.

### Relationship between different foci

To study nuclear foci during the transition of viral replication through different stages of infection we have studied three types of nuclear foci. The first, formed by the E1 and E2 proteins, recruit Brd4 and DNA damage response markers, but do not contain viral DNA and so cannot progress to full-blown replication foci. We hypothesize that these stalled nuclear foci mimic immature nuclear foci that cannot amplify viral DNA because of the cellular environment and/or limiting viral proteins in a real infection. Viral foci formed with E1, viral DNA and a DNA-binding defective E2 protein are stalled at a similar stage confirming that a competent replication initiation complex is required for transition to the next step. The second type of foci contains E1, E2 and a viral origin-containing replicon. These foci expand, displace Brd4 but do not need Brd4 for their formation. This seems counter-intuitive if we are proposing that they arise from the first type of foci. However, to generate these foci we must transfect viral DNA along with E1 and E2 expression vectors and so we have likely bypassed the requirement for the initial establishment phase. The third type of foci is the most authentic; they form in differentiated cells derived from clinical lesions, express other viral proteins and contain the complete HPV genome. Yet, these foci share many characteristics of the E1/E2/ori replication foci, and appear to expand from adjacent foci of Brd4. While these experimental settings can only attempt to mimic the different stages of HPV replication, they allow us to dissect the requirements for each stage.

### Models

Although the function of Brd4 in viral transcriptional regulation is well understood, its precise role in the development of viral replication foci remains elusive. We have previously proposed that Brd4 tethers the viral genome to host chromatin at early stages of infection to regulate viral transcription and ensure that the genome is located in active regions of the nucleus [35]. We propose that this positioning might also enhance the establishment of replication foci and Brd4 might help nucleate DNA replication on host chromatin by recruiting E1, E2, and the viral genome to regions of the host genome that are susceptible to replication fork stalling, DNA damage and late replication. In uninfected cells, damage to cellular DNA results in a huge influx of repair factors to nuclear foci, and long stretches of surrounding chromatin are modified by γH2AX. In infected cells, attachment of the viral genome-E1/E2 complex to susceptible chromatin would allow the virus to take advantage of this cascade. However, as the viral genomes replicate and the foci grow larger, we would predict that Brd4 is no longer necessary and is displaced to the perimeter of the growing foci. This implies that Brd4 has no direct role in replication but can enhance it by associating with permissive regions of the nucleus.

This association of viral genomes with susceptible host chromatin could occur early in infection when genomes are being established, during maintenance replication, or at the transition between maintenance replication and vegetative replication in differentiating cells. HPVs induce the ATR response in foci during the initial amplification phase of infection [5] but expression and nuclear localization of the E1 protein must be tightly regulated to allow persistent viral replication and cell division [36]. In differentiated cells, the ATM pathway enhances amplificational viral genome replication [9]. Furthermore, HPVs amplify their genomes in cells that have completed cellular DNA replication and are in the G2 phase, or have exited the cell cycle [37], [38]. Thus, tethering genomes to regions of the host chromatin that replicate late in the cell cycle, and are susceptible to DNA damage is an effective strategy. Papillomaviruses could take advantage of the DNA repair machinery, and use this to enhance viral DNA amplification in differentiated cells. This would allow papillomaviruses to amplify viral DNA in differentiated cells without competition from host DNA synthesis and would concentrate viral replication in nuclear factories.

An alternative or compatible model is that Brd4 is protecting adjacent cellular chromatin from an ATM kinase signaling cascade resulting from viral DNA replication or replication proteins [39], [40]. Floyd et al. recently demonstrated that the B isoform of Brd4 (an N-terminal form) functions to limit the ATM signaling storm resulting from DNA damage [39], [40]. The observed localization of Brd4 in the three types of replication foci is consistent with this role: Brd4 colocalizes with E1/E2 foci that form and promote an ATM response on host chromatin; however, it is dispersed, or forms a peripheral pattern on foci that are replicating viral DNA and presumably displacing cellular DNA. The location of Brd4 in each of these cases corresponds to the presumed location of host chromatin. Notably, the adjacent/satellite pattern of Brd4 observed next to HPV16 and HPV18 replication foci (Figure 11D) was observed much more readily with an antibody directed against the N-terminal half of Brd4 compared to a C-terminal Brd4 antibody (data not shown). Future studies will address which Brd4 isoform is recruited to HPV replication foci. The observed localization of Brd4 could represent the cell's response to restrict the DNA damage response to the viral replication foci and protect adjacent host chromatin. However, it would also not be surprising if the virus took advantage of this response to promote the viral life cycle.

### Association with common fragile sites

In a complementary study in our laboratory we have identified the regions of the human genome bound by the HPV1 E2 and Brd4 proteins, a complex that binds tightly to host chromatin. We find that the HPV1 E2/Brd4 complex, as well as an HPV16 E1/E2 complex, binds to fragile sites of the human genome in C-33A cells. We find that cancer-derived cells (C-33A and other) often contain Brd4 binding regions that extend over large stretches of DNA (>1 Mb) and are visualized as prominent nuclear foci (Jang, Shen, McBride, manuscript submitted). These fragile sites are often located adjacent to the Brd4 foci associated with 9E replication foci. Notably, in cancers HPVs are often integrated close to fragile sites [41] and this accidental integration event could result from the close association of replication foci with fragile sites. Furthermore, one of the characteristics of fragile sites is that they often replicate very late in the cell cycle [42].

Brd4 binding to fragile sites can extend to over a megabase region of the host genome and we believe that as cells become progressively more genetically unstable, these fragile regions become more and more prominent (Jang, Shen, McBride, manuscript submitted). In HPV31 9E cells (derived from a clinical lesion) Brd4 is very frequently found adjacent to small replication foci, or in a satellite pattern around large replication foci. However, this pattern is not prominent in HPV31 cell lines newly established in HFKs (see Figure 11). Taken together with the other studies from our laboratory described above, we believe that the prominent Brd4 foci observed around viral replication foci in clinically derived cell lines such as 9E could reflect increased genetic instability of these regions. It is difficult to determine from the experiments presented here whether the replication foci that form in newly established lines are adjacent to small Brd4 foci or are independent of this association. However, as shown in Figure 11D, adjacent/satellite Brd4 foci are often found adjacent to viral replication in other HPV containing cell lines. Each of these lines harbors an oncogenic HPV that will promote genetic instability and augments expression of these sites, already susceptible to replication stress. We propose that the Brd4 foci become more prominent with increased genomic instability. This could present an attractive target to a virus that can enhance amplification of its genome by inducing the cellular DNA damage response or could represent the cells attempt to limit the ATM DNA damage cascade.

### Evidence for a switch in E2 function from transcription to replication mode

The E2 protein is important for transcriptional regulation and viral DNA replication. A couple of studies indicate that the transcriptional function of E2 is not required for late stages of viral replication. Alderborn et al. demonstrated that BPV1 encoding a temperature sensitive E2 protein that could support replication but not transcription at the non-permissive temperature could amplify viral DNA in growth-arrested mouse cells (a system thought to mimic induction of vegetative BPV-1 DNA synthesis in productively infected warts [43]). Stubenrauch and colleagues showed that HPV31 genomes encoding E2 proteins mutated in residues that are crucial for transcriptional regulation were also able to support late replication functions [33]. Therefore, it appears that there is a switch from an E2-dependent transcriptional mode to a replication mode at later times of viral infection. To date, it has not been possible to separate E2 transcriptional functions from Brd4 binding and this would imply that Brd4 binding is not required for late functions of the E2 protein.

Several groups have shown that the ability of E2 to bind Brd4 is not required for E1–E2 mediated transient replication [13], [44]–[46]. Stubenrauch et al. have also shown that HPV31 genomes with E2 mutations in R37 and I73 in HPV31 E2 (later shown to abrogate Brd4 binding [12]) could replicate extrachromosomally and the genome containing the I73L mutation could also amplify its genome in the differentiated cells of organotypic raft cultures, although amplification and late gene expression were reduced [33]. We have generated keratinocyte lines containing these genomes and reproduced the ability of these viruses to replicate (Figure 11); we find that they also generate replication centers in differentiated cells. This fits quite well with our hypothesis that one role of Brd4 is to assist in the nucleation of viral replication foci at sites in the host that are most conducive for induction of the DNA damage response and recruitment of the replication machinery, but is not absolutely required.

### E1, E2 and Brd4 protein interactions

The E2 proteins of BPV1 tether the viral genome to host chromosomes in complex with the chromatin binding Brd4 proteins [11], [13], [47], and the E2 proteins from several papillomaviruses bind in a similar fashion to host chromosomes [14], [19]. However, the alpha-papillomavirus E2 proteins are not easily detected on mitotic chromosomes [19] and do not bind tightly to host chromatin with Brd4 [14]. However, as shown here, when expressed along with the E1 protein, E2 and Brd4 from this genus of viruses colocalize in nuclear foci on host chromatin. Because expression of E1 induces a DNA damage response and growth arrest [4], [6], we were not able to study whether the E1–E2-Brd4 complex bound mitotic chromosomes. However, it is possible that in the context of a viral infection other viral proteins could suppress this growth arrest and allow division of cells with E1/E2 bound to chromatin.

The crystal structure of the HPV18 E2 transactivation domain has been solved in complex with either a C-terminal domain of the E1 protein [48] or with a C-terminal peptide of Brd4 [49]. E1 and Brd4 interact with opposing faces of the E2 transactivation domain and so it is, at least theoretically, possible that all three proteins could form a complex. Using a Brd4-E1–E2 binding assay described previously [13], we find that all three proteins can form a complex (data not shown). In our previous studies we had observed that the alpha-HPV E2 proteins did not colocalize with Brd4 in vivo and also demonstrated weak E2-Brd4 binding when compared to other E2 proteins [14]. We wondered whether the presence of the E1 protein might increase binding between E2 and Brd4, perhaps by inducing a conformational change in the transactivation domain. However, we did not observe any increase in the binding capacity of E2 and Brd4 in the presence of E1, at least under the conditions tested (data not shown). In accordance with this observation, while E1 can increase the association of E2 with chromatin, Brd4 is still easily eluted from the nucleus (Figure 2).

It is possible that other regions of E2 (and E1) might interact with Brd4 to stabilize the complex in nuclear foci. Although most studies focus on the interaction between the CTD of Brd4 and the R37 and I73 residues in the E2 transactivation domain, it is likely that there are additional interactions. For example, HPV11 E2 interacts with an N-terminal region of Brd4 in addition to the CTD, implying that there must be additional Brd4 interaction regions on the E2 protein [31]. Brd4 can also undergo a phosphorylation-mediated conformational switch that modulates its interaction with associated proteins [50]. Brd4 is also recruited to Merkel polyoma virus (MCPyV) replication foci [51]; and replication of MCPyV is dependent on T antigen, a protein analogous to E1. Therefore, E1 might also play a role in the recruitment of Brd4 to the nuclear foci. There is also evidence that Brd4 may also play an E2-independent role in viral replication [46]. Ilves et al. have shown that the C-terminal domain of Brd4 (which interacts with E2) can inhibit both papillomavirus and polyoma virus replication in an E2 independent manner (i.e. an R37/I73 E2 mutation) [46].

### Characteristics of chromatin in HPV replication foci

Since Brd4 is a chromatin adaptor protein that binds acetylated histones, we examined histone marks in chromatin associated with each type of nuclear or replication focus. The foci formed by E1–E2 proteins always co-localized with Brd4 and were highly enriched in acetylated histones. These foci induce the cellular DNA damage response and recruit many markers of the ATM/ATR pathways [4]–[6]. Cellular repair responses require open chromatin consistent with histone acetylation, and Brd4 is known to decompact chromatin [52]. Foci do not form in the absence of the DNA damage response, which is induced by the E1 DNA binding and helicase functions [4]. Furthermore, E2 can associate with CBP/p300, which can further acetylate local chromatin and enhance Brd4 binding (reviewed in [53]). We hypothesize that, in these foci, E2 functions in a “transcriptional” mode. However, as the foci begin to amplify viral genomes the acetylated chromatin marks are dispersed to the periphery of the foci, or are dissipated. It is possible that as the replication foci grow, cellular DNA is displaced to the periphery, along with acetylated histones and Brd4.

### Evidence for homologous recombination in HPV replication

There is evidence that there is a switch in the mode of viral replication at late times of infection [43], [54]. Initially it was proposed that replication switched from a bidirectional theta mode to a rolling circle mode [54]. However, recent findings that late differentiation-dependent genome amplification is enhanced by the DNA damage response pathways [9] implicate DNA repair pathways in late viral replication [4], [8], [10]. The two major DNA repair pathways in higher eukaryotes are non-homologous end joining (NHEJ) and homologous recombination (HR), however only the latter is of high fidelity. Many viruses use a variation of the homologous recombination pathway to replicate their genomes at late times in infection (reviewed in [8]) and it is likely that HPVs use this mechanism rather than the classical rolling circle mode. The classical marker of HR (at least in human cells) is Rad51, a recombinase that promotes strand invasion and homologous pairing. We show here that Rad51 localizes to the core of large replication foci in differentiated HPV31 containing cells (also shown by Gillespie et al. [10]), consistent with a switch to a recombination-directed replication (RDR) mode. However, in the presence of a viral replicon, the E1 and E2 proteins are capable of inducing replication foci that contain Rad51. Therefore, other viral proteins are not required for the switch to an RDR mode of replication.

Although the E1 and E2 proteins are sufficient for the formation of replication foci, and for the recruitment of Rad51, it is unlikely that they are sufficient to support late replication in the complete viral life-cycle. Both E6 and E7 functions are required for maintenance replication of the viral genomes [55]–[57] and E7 promotes late replication by binding to ATM and enhancing the DNA damage response in differentiated cell [9] and by activating STAT-5, which leads to induction of ATM phosphorylation through the PPARγ pathway [58]. We believe the ability of E7 to induce replication stress [59] might also enhance nucleation of viral replication foci on host chromatin at early times in infection and contribute to persistent infection.

### Comparison with other studies analyzing the role of Brd4 in replication

During the preparation of this manuscript, Wang et al., published a study showing that Brd4 is recruited to E1/E2 replication foci in C-33A cells and that this interaction is crucial for HPV replication [60]. Our findings are somewhat different since we find that, while Brd4 is co-localized with and is essential for the formation of E1/E2 foci on host chromatin, it is not essential for the development of actively replicating E1/E2 foci or virus generated replication centers in keratinocytes. One possible difference that might reconcile these findings is the cell type used. The studies presented here were carried out in primary keratinocytes while Wang and colleagues used C-33A cells [60]. C-33A cells are derived from an HPV negative cervical carcinoma and are genetically unstable. Even in the absence of HPV proteins, the Brd4 protein is bound to specific regions of the genome, which we have shown to be fragile sites (Jang, Shen, McBride, manuscript submitted). We have also previously noted that the E1 protein can form foci in these cells in the absence of E2, which could result in different localization and requirements for Brd4.

### Brd4 and cellular DNA damage

Brd4 is recruited to E1–E2 foci undergoing a DNA damage response and we wondered whether Brd4 could play a role in the cellular DNA damage and repair response. To test this, cellular DNA was damaged in a number of different ways (UV damage, γ-irradiation, aphidicolin, hydroxyurea) and examined for specific Brd4 localization. However, we were unable to detect Brd4 recruited to repair foci or co-localized with any DNA damage response proteins after these treatments (data not shown). Nevertheless, we feel that there is still a strong likelihood that Brd4 is in some way involved in cellular replication and/or repair. Brd4 forms a complex with RFC [61], a factor that loads PCNA onto the replication fork and with SMC5/6, structural maintenance of chromosome complexes that are frequently associated with repair and recombination and is found in complex with E2 [31]. The alternative RFC1 subunit, ATAD5, which is involved in the DNA damage response, binds to the ET domain of Brd4 [62]. As discussed above, it has recently been demonstrated that the B isoform of Brd4 limits the ATM response to protect cellular chromatin [39]. Persistent viruses such as papillomaviruses, often usurp key cellular processes for their own needs and further studies of Brd4's role in viral DNA replication could provide insight into its role in cellular DNA damage response and repair pathways.

### The E2-Brd4 interaction is highly conserved

The E2-Brd4 interaction must play an important role at some stage of the viral life cycle since E2 residues R37 and I73 are conserved in virtually all papillomavirus genomes that have been isolated to date [63]. These residues are usually considered to be important for both the transcriptional activation and repression functions of E2 [13], [31], [64]. In the alpha-PVs, E2 is thought to function primarily as a transcriptional repressor [65] and this function is mediated by Brd4 [31], [66]. Alleviation of repression by mutation of the residues that interact with Brd4 might not be completely detrimental to the viral life cycle when studied in a cell culture system, as was found by Stubenrauch et al. [33]. Alleviation of the E2 transcriptional repression function might counteract any requirement for the E2-Brd4 interaction to enhance viral replication. Keratinocyte cell lines studied in the laboratory usually maintain high copies of the viral genome that can be induced to differentiate to measure late replication and gene expression. Alternatively, they are obtained by DNA transfection, which might bypass the early stages of genome establishment. These cell lines may not reflect the true life situation where very low copy number of viral genomes are established, replicated and maintained in the basal cells of a papilloma [67] and are greatly induced to amplify upon differentiation [22]. In these circumstances it would be important to have a highly efficient process to recruit DNA synthesis machinery to the regions of the nucleus that harbored the viral genomes.

## Materials and Methods

### Plasmids

HPV16 E2 proteins, tagged with an N-terminal FLAG epitope and expressed from the metallothionein inducible promoter in the pMEP4 plasmid, and mutations therein, have been described previously [38]. The HPV16 E1 genes with N-terminal glu-glu (EE) epitope tags, and mutations therein, have been described previously [4]. Mutations were introduced into HPV16 E2 to generate amino acid substitutions R37K and I73L, using GeneArt (Invitrogen). The origin plasmid, p16ori containing HPV16 nucleotides 7838-130 cloned in a pKS Bluescript vector has been described previously [68]. The empty vector, pKS was used as a control. The HPV18 genome, cloned in pBR322, has been described previously [69].

### Recircularized HPV genomes

To generate recircularized HPV genomes, the genomes was liberated from the vector by restriction digestion and religated at 5 µg/ml to enhance intramolecular ligation. HPV31 genomes with mutations in E2 residues I73L and R37K were described previously [33].

### Cell culture

Human foreskin keratinocytes (HFK) were grown in F-medium (3∶1 [v/v] F-12 [Ham]-DMEM, 5% FBS, 0.4 µg/ml hydrocortisone, 5 µg/ml insulin, 8.4 ng/ml cholera toxin, 10 ng/ml EGF, 24 µg/ml adenine, 100 U/ml penicillin, and 100 µg/ml streptomycin) and 10 µM Y-27632 in the presence of irradiated 3T3-J2 feeder cells as described [70]. HPV containing cell lines (CIN-612 9E, W12 20863, B1 HPV18 cells harboring HPV genomes and HPV31 wildtype (WT), R37K and I73L genomes) were grown in F-medium in co-culture with irradiated 3T3 feeders.

### Generation of keratinocyte cell lines with replicating HPV genomes

Primary HFKs were electroporated with recircularized HPV genomes using an Amaxa nucleofector. The cells were were cotransfected with pRSVneo, and underwent a four day selection in 200 µg/ml G418 selection. Resulting colonies were pooled, passed several times and cultured for experiments.

### Differentiation of keratinocytes with calcium

Cell lines and HFKs were differentiated with calcium, essentially as described previously [9]. Feeders and keratinocytes were seeded as described above. When 90% confluent, the medium was changed to Lonza Growth medium (KBM plus supplement media). Twenty four hours later, the medium was changed to Differentiation medium (Lonza KBM/1.5 mM CaCl_2_/no supplements). Cells were cultured for the times indicated before harvest or fixation.

### Transient transfection and immunofluoresence

Cells were seeded on glass coverslips or slides and cultured with irradiated NIH-3T3 J2 feeders in F-media containing 10 µM Y-27632. Transfections were performed using FuGENE 6 (Roche) with 0.4 µg each DNA (0.8 µg total) for each well of a 12 well plate using a DNA: FuGENE ratio of 1∶3 (µg∶µl) as recommended by the manufacturer. Where noted, 25 or 50 ng plasmids containing the HPV16 minimal origin of replication p16ori (or control plasmid pKS) were added to the transfection. E1 and E2 protein expression was induced with 3 µM CdSO_4_ induction for 4 hours prior to fixation with 4% paraformaldehyde at 24–28 hours post transfection. Cells were permeabilized in 0.1% triton X-100 for 15 min and blocked in 0.25% BSA/gelatin in PBS. Cells were stained with primary antibody for 1 hour at 37°C and were stained with DyLight secondary antibodies (Jackson ImmunoResearch) for 30 min at 37°C. The slides were mounted with DAPI containing Prolong Gold (Invitrogen). Images were collected on a Leica TCS-NT/SP5 confocal microscope (Leica Microsystems) using a 40× or 63× oil immersion objective NA 1.4. Images were processed using Leica AS Lite software, or Bitplane Imaris software (Zurich, Switzerland) or were deconvolved with Huygens Essential software (Scientific Volume Imaging B.V., VB Hilversum, Netherlands), where indicated.

### Antibodies

Antibodies used were against EE (chicken polyclonal, Bethyl laboratories, Inc, A190-109A; 1∶100); FLAG (M2 mouse monoclonal, Sigma; 1∶500); phospho- Ser1981 ATM (Abcam ab81292; 1∶100); phospho-Histone H2AX (Ser139), mouse IgG1, Millipore 05-636, 1∶500); Rad51 (mouse IgG2b; Abcam ab213, 1∶500); Rad51 (goat polyclonal, Santa Cruz sc-6862, 1∶50); H4K8ac, rabbit polyclonal, Upstate Biotechnology 07-328, 1∶500); H3K56ac, rabbit polyclonal, Epitomics 2134-1, 1∶500), H3K4me1, rabbit polyclonal, Abcam 2134-1, 1∶200), tubulin (mouse monoclonal, Sigma T5168, 1∶10,000). The C-terminal specific Brd4 antisera was raised in rabbits against a C-terminal peptide of Brd4 and affinity purified (MCB2; 1∶100). The N-terminal directed rabbit polyclonal antiserum, 2290 (used at 1∶200), was a gift from Keiko Ozato and was described previously [18]. The 8H2 mouse monoclonal antibody recognizing amino acids 287–530 of human Brd4 was generated at UT Southwestern Antibody Core Facility by using recombinant FLAG-tagged full-length human Brd4, purified from insect Sf9 cells [16], as the antigen. Screening of hybridomas was first conducted by ELISA and then validated by Western blotting using FLAG-tagged human Brd4 protein fragments spanning aa 1–530, 287–530, 1–722, 598–785, 721–1055, and 1110–1362, respectively, purified from bacteria as described [50].

### Brd4 inhibitors

Brd4 inhibitors GSK525762A525762A and its enantiomer were synthesized as described previously [71] from 2-amino-5-methoxybenzoic acid. They were prepared as racemic mixtures, which were separated with Chiralcel® OD-H column. GSK525762A525762A [α]_D_ = +84(C = 0.075/MeOH, 95% ee), the enantiomer [α]_D_ = −87(C = 0.075/MeOH, 99% ee). (±)-JQ1 was prepared as racemic mixtures as described [27]. Cells were treated for 24 hours before transfection of HPV16 E1 and E2 plasmids with 2 µM GSK525762A+/−, 0.5 µM JQ1, or a DMSO solvent control. About 20–100 cells expressing the E1 protein were counted in each experiment and scored for focus formation.

### siRNA treatment

Cells were treated with FlexiTube siRNA GeneSolution GS23476 for Brd4 (Qiagen) or control siRNA for 24 hours before transfection with HPV16 E1 and E2 plasmids. Cells were cultured for an additional 24 hours before fixation or harvest. About 20–100 cells expressing the E1 protein were counted in each experiment and scored for focus formation.

### In vitro Brd4 binding experiment

HPV16 E1 and E2 proteins were synthesized using TNT® Coupled Reticulocyte Lysate System (Promega). Purified FLAG-tagged Brd4, described previously [13] was incubated with ^35^S-labeled E1 and E2 proteins in PBS/10% glycerol/0.1% triton X-100/10 mM MgCl2/1 mM DTT, and isolated with anti-FLAG agarose beads. Complexes were washed, eluted, separated by SDS-PAGE and visualized with autoradiography.

### Western blotting

Cells were lysed in ice-cold RIPA buffer (20 mM Hepes pH 7.3, 150 mM NaCl, 1 mM EDTA, 1% NP-40, 1% sodium deoxycholate and 0.1% SDS) containing protease inhibitor Complete and PhosSTOP tablets (Roche). Equal amounts of total protein (6–15 µg per lane) were separated in NuPAGE Bis-Tris gels (Invitrogen), as recommended by the manufacturer. The proteins were transferred to Immobilon P membrane (Millipore) and specific proteins were detected with mouse monoclonal anti-EE for E1 (1∶4 dilution), M2 anti-FLAG (Sigma; 1∶10,000 dilution) for E2, rabbit Brd4 (mcb2; 1∶2000) and mouse anti-tubulin (1∶10,000), which were detected with appropriate secondary antibodies. Protein bands were detected with Super Signal Dura (Thermo scientific) and images were captured digitally using a Kodak Imagestation or by autoradiography.

### Southern blotting

Total cellular DNA was prepared from HPV containing cells using the Qiagen DNeasy Blood and Tissue kit. 2 µg DNA was digested with an enzyme that linearized the viral genome (HindII). Digested and undigested DNA samples were separated on an agarose gel and transferred to nylon membranes using a TurboBlotter system (Whatman/S&S). Viral DNA was detected by hybridization with ^32^P-labeled HPV31 DNA generated using the Roche Random Prime kit and detected using a Typhoon Imager (Molecular Dynamics).

### Fluorescent in situ hybridization (FISH) and combined immunofluoresence-FISH

9E cells were cultured on coverslips and differentiated for five days as described above. The cells were fixed with cold methanol∶acetic acid (3∶1) for 15 min and the methanol∶acetic acid fixation steps repeated two more times. The cells were treated with RNace-It (Stratagene) for 1 hour, dehydrated with 70%, 85%, and 100% ethanol, and dried for several hours. The HPV31 probe was prepared using ULysis nucleic acid labeling kit (Molecular Probes), purified through illustra ProbeQuant G-50 micro-column (GE Healthcare), and resuspended in TE containing 0.3 µg/µl of Cot-1 DNA. Alexa 488-labeled HPV31 genome (50 ng) was mixed with 8 µl FISH hybridization buffer (Empire Genomics), applied to the cells on coverslip, denatured at 75°C for 5 min and hybridized overnight at 37°C. Cells were washed in 1× phosphate-buffered detergent (Qbiogene) for 5 min at room temperature, 1× wash buffer (0.5× SSC, 0.1% SDS) for 5 min at 65°C, and 1× phosphate-buffered detergent for 5 min at room temperature. Cellular DNA was stained with DAPI.

For combined immunofluorescence and FISH, cells were fixed with 4% PFA as described for Immunofluorescence, and antibody staining was carried out as described. The stained cells were subsequently treated with methanol∶acetic acid (3∶1) for 10 min and 2% PFA for 1 min and dehydrated with sequential treatments of 70%, 85% and 100% ethanol for 3 min. FISH was performed as described above. For the HPV16 origin probe, nucleotides 7458-122 of the HPV16 genome were PCR amplified. Purified DNA fragments were labeled with Alexa 594 using ULysis nucleic acid labeling kits and purified using Illustra ProbeQuant G-50 microcolumns. Following ethanol precipitation, labeled HPV16 origin probe (1 µg) was dissolved in 10 µl of TE buffer containing 3 µg of Cot-1 DNA. Cells were washed as described above. Images were collected using a Leica TCS-SP5 laser scanning confocal imaging system.
